# Association of Severity of *Helicobacter pylori* Infection with Peripheral Blood Neutrophil to Lymphocyte Ratio and Mean Platelet Volume

**DOI:** 10.5005/jp-journals-10018-1204

**Published:** 2017-05-05

**Authors:** Mustafa Guclu, A Faruq Agan

**Affiliations:** 1 Department of Gastroenterology, Istanbul Medipol University, Istanbul, Turkey; 2 Department of Gastroenterology, Istanbul Medipol University, Istanbul, Turkey

**Keywords:** *Helicobacter pylori*, Mean platelet volume, Neutrophil to lymphocyte ratio.

## Abstract

**Aim:**

To determine the correlation of *Helicobacter pylori* infection with peripheral blood neutrophil/lymphocyte ratio (NLR) and mean platelet volume (MPV).

**Materials and methods:**

The NLR, MPV, platelets, leukocytes, neutrophils, and lymphocytes were calculated and the differences between groups were investigated.

**Results:**

A total of 199 patients were included in the study. Neutrophil/lymphocyte ratio was statistically lower in *H. pylori-positive* patients than in *H. pylori-negative* patients (1.94 ± 0.79 *vs* 2.67 ± 2.35 respectively, p = 0.04). There was no significant difference between *H. pylori-negative* patients and *H. pylori-positive* patients of severe intensity in terms of MPV. However, peripheral blood lymphocytes and platelets were statistically significantly higher in *H. pylori-positive* patients of severe intensity (lymphocytes 2150 ± 826 *vs* 2954 ± 2436 respectively, p = 0.000 and platelets 258247 ± 69494 *vs* 265611 ± 113397 respectively, p = 0.02) compared with *H. pylori-*negative patients.

**Conclusion:**

A moderate increase in the intensity of *H. pylori* does not lead to a significant change in MPV as measured by hemogram; however, it gives rise to a statistically significant fall in NLR. Presence of severe *H. pylori-positive* intensity leads to a statistically significant increase in peripheral blood lymphocytes and platelets compared with *H. pylori-negative* patients.

**How to cite this article:** Guclu M, Agan AF. Association of Severity of *Helicobacter pylori* Infection with Peripheral Blood Neutrophil to Lymphocyte Ratio and Mean Platelet Volume. Euroasian J Hepato-Gastroenterol 2017;7(1):11-16.

## INTRODUCTION

*H. pylori* is the most common infectious disease in the world.^1^ More than half of the world’s population has *H. pylori* colonization.^2^ It is a widespread microorganism encountered at a frequency of 80% in developing countries and 20 to 50% in Western societies.^1^ *H. pylori* causes local inflammation in the stomach and systemic humoral immune response. The majority of the cases have an asymptomatic, chronic inflammation.^3^ *H. pylori* infection, which has a high rate of morbidity, is recognized as a worldwide problem and the most frequent cause of chronic gastritis.^4^ The International Agency for Research on Cancer reported *H. pylori* as a class 1 carcinogen in humans in 1994.^5^ *H. pylori* is inclined to settle irregularly in the gastric antrum as this is a less acidic medium. *H. pylori* attracts neutrophils and lymphocytes with several chemotactic proteins released in the stomach. Some substances secreted by mononuclear cells and neutrophils induce mucosal inflammation and thus cause gastritis. In conclusion, the gastric mucosa is infiltrated by neutrophils, macrophages, and lymphocytes in addition to several signal cytokines, and a subclinical systemic, low-grade inflammation occurs. *H. pylori* is particularly associated with severe gastric diseases like chronic gastritis, peptic ulcer, gastric lymphoma, and gastric cancer.^2,3^ In Japan, it was discovered that serum interleukin-6 (IL-6) was significantly high in patients with high serum anti-H. *pylori* levels.^6^ Interleukin-6 is a multifunctional cytokine secreted from numerous cells, including notably, mono-cytes, lymphocytes, mesangial cells, and endothelial cells. Some epidemiological studies have shown that there is a direct correlation between serum IL-6 level and severity of coronary artery disease.^7,8^ For this reason, they emphasized that the presence of *H. pylori* has a very important role in other extragastroenterologic diseases like cardiovascular diseases, and induces systemic inflammation due to elevation of IL-6. Due to chronic inflammation that it stimulates, *H. pylori* is able to create persistent antigenic stimulation, thus causing a systemic inflammatory reaction. On the contrary, the increase in C-reactive protein (CRP) is correlated with endothelial dysfunction, and it has been demonstrated that the presence of *H. pylori* infection elevates CRP in blood.^9^ C-reactive protein is a hepatic acute-phase reactant whose synthesis is regulated by IL-6.^10^ *H. pylori* stimulates production of proinflam-matory cytokines like tumor necrosis factor (TNF), IL-1, IL-6, and IL-8.^11^ In persons infected with *H. pylori,* TNF, IL-1, IL-6, and IL-8 serum levels are found to be high.^12^ C-reactive protein production is influenced by hormones, such as cortisol, mainly ILs.^13^ Therefore, there is interaction and correlation between *H. pylori* and acute-phase reactants like CRP and ILs.

It is asserted that reduction in platelets occurs as a result of antibodies to *H. pylori* reacting with platelet glycoproteins, and, as a result, idiopathic thrombocy-topenic purpura (ITP) develops. In patients with ITP, platelet counts increase due to *H. pylori* eradication and autoantibodies disappear in majority of the sub-jects.^14^ Platelet count and functions play a key role in cardiovascular events. Mean platelet volume (MPV) is an index showing platelet volume. High MPV values show larger and very active platelets, which contribute to thrombocytic events. Some studies show that, in cases, such as diabetes mellitus and coronary artery disease, where low-grade inflammation exists, these patients have higher MPV values.^15^

As the physiological response of leukocytes in circulation to stress causes an increase in neutrophil count and a decrease in lymphocyte count, the proportion of these two subgroups to each other is used as a sensitive marker of inflammation. Changes occur in rates of leukocytes in circulation during the inflammatory response. Neutro-philia is accompanied by relative lymphopenia. Neutro-phil to lymphocyte ratio (NLR) is obtained from a simple hemogram and is an inflammatory marker of various diseases. Increased NLR was found to be an indicator of bad prognosis in patients undergoing cardiovascular intervention, and recently, it was shown that mortality rate increased in acute coronary syndromes with the elevation of NLR.^16^ When evaluated with sepsis scores, such as Acute Physiology and Chronic Health Evaluation II (APACHE 2) and Sepsis-related Organ Failure Assessment (SOFA), this ratio was found to be consistent with the severity and prognosis of the disease, and was named as the neutrophil-lymphocyte stress factor. These NLR and MPV in peripheral blood are used as a parameter that provides information about the correlation between the inflammatory medium and physiological stress.

## MATERIALS AND METHODS

This study was performed with the approval of the ethics board of Istanbul Medipol University Faculty of Medicine dated January 23, 2015 Nr.10840098-19 and 15. A total of 199 prospective patients, consistent with the study criteria, who applied to the Istanbul Medipol University, Gastroenterology polyclinic, during the period between January 2015 and September 2015 have been included in the study after having obtained their consent.

In this study, 199 patients with only dyspeptic complaints with no other disease, and with antral gastritis on gastroscopy were enrolled. Only from patients with antral gastritis, two biopsies were endoscopically obtained from the antrum, and all biopsies were evaluated histopatho-logically according to Sydney classification.^17^ Patients with atrophy and intestinal metaplasia according to Sydney classification were excluded and only patients with antral gastritis were included in the study. Patients with additional pathology other than antral gastritis were excluded. *H. pylori* charges were identified as mild, moderate, and severe *H. pylori* intensity. Patients with gastritis were split into four groups as *H. pylori*-negative and *H.* pylori-positive according to intensity (mild, moderate, and severe). Hemogram tests were performed for all patients. The NLR, MPV, platelets, leukocytes, neutrophils, and lymphocytes were calculated by a simple hemogram test and the differences between these four groups were investigated; NLR was calculated by dividing absolute neutrophil count by absolute lymphocyte count as measured by hemogram.

### Exclusion Criteria

Patients with (1) gastroscopy findings other than antral gastritis, (2) gastrointestinal hemorrhage, (3) gastric and duodenal ulcer, (4) portal hypertension, (5) diabetes mellitus, (6) systemic diseases and chronic diseases, and (7) nonsteroidal anti-inflammatory, proton pump inhibitors, and cytotoxic medicines were excluded from the study.

### Histopathologic Evaluation

Mucosal samples of all patients were stained with hema-toxylin and eosin. Histopathologic parameters were determined according to Sydney system. Hematoxylin-eosin-stained sections were evaluated for the presence of *H. pylori* by Sydney classification.

### Biochemical Measurements

Samples were collected from the antecubital vein into ethylenediaminetetraacetic acid (EDTA)-containing vacuum tubes (15% K3 EDTA 0.054 mL/4.5 mL blood) for hemogram and automated blood count, and the samples obtained were analyzed in 1 hour. Hemogram tests were performed in automatic complete blood count analyzer (A Sysmex XE-2100, Sysmex Corporation, Kobe, Kansai, Japan). Normal laboratory reference values were as follows: MPV: 8 to 12 fL, platelet: 150,000 to 450,000/μL, leukocyte: 3,500 to 11,000/μL, neutrophil: 1,800 to 7,920/μL, lymphocyte: 1,500 to 4,000/μL.

### Statistical Analysis

The patients who complied with the study criteria and applied to the Istanbul Medipol University Gastroen-terology Polyclinic during the period between January 2015 and September 2015 were consecutively admitted into the study. A total of 199 prospective patients were included in the study after having obtained their consent. This is a prospective and single-center study. Statistical Package for the Social Sciences (SPSS) for Windows 21.0 version (SPSS for Windows 21.0, Chicago, Illinois, USA) was used for statistical study and analysis of data. The differences between the groups were compared using chi-square and Student’s t-test. Subgroup analyses were made among the mild, moderate, and severe *H. pylori* patients. Statistical analysis included parametric tests, nonparametric tests of comparison, and one-way analysis of variance. Results were presented as mean ± standard deviation (SD) and a value of p < 0.05 was considered as statistically significant.

## RESULTS

A total 199 patients, 88 males and 111 females, who had only antral gastritis on endoscopy, were included in the study. A total of 101 *H. pylori-positive* patients with a mean age of 48 ± 17 years, comprising 54 females and 47 males, and a total of 98 *H. pylori*-negative patients with a mean age of 45 ± 16 years, comprising 57 females and 41 males, were enrolled in the study. No significant difference was identified between the groups in terms of mean age and gender. The distribution of this parameter, in which 199 patients enrolled in the study of *H. pylori*-negative and *H. pylori*-positive patients are compared, is presented in Table 1.

There were no differences in terms of NLR and MPV between total *H. pylori*-negative patients and *H. pylori-*positive patients. There were no differences in terms of NLR and MPV between *H. pylori*-negative patients and 57 *H. pylori*-positive patients of mild intensity. There was no difference between *H. pylori*-negative patients and 26 *H. pylori*-positive patients of moderate intensity in terms of MPV (10.18 ± 0.91 *vs* 9.84 ± 0.77 respectively, p = 0.39), whereas NLR was statistically lower in *H. pylori-positive* patients than in *H. pylori*-negative patients (1.94 ± 0.79 *vs* 2.67 ± 2.35 respectively, p = 0.04). While there was no significant difference between *H. pylori-negative* patients and 18 *H. pylori*-positive patients of severe intensity in terms of MPV and NLR, peripheral blood lymphocytes were statistically significantly higher in *H. pylori-positive* patients of severe intensity (2150 ± 826 *vs* 2954 ± 2436 respectively, p = 0.0001, normal laboratory lymphocyte reference range: 1,500-4,000). There was no statistically significant difference between the groups in terms of peripheral blood leukocytes and neutrophil counts; other parameters tested in hemogram and compared. No significant difference was noted between the groups in terms of MPV. However, in the comparison of these four groups, statistically higher platelet counts were found in *H. pylori*-positive patients of severe intensity than in *H. pylori*-negative patients on hemogram (p = 0.02).

**Table 1:** **Table 1:** Comparison of 98 *H. pylori-negative* and 101 *H. pylori-positive* patients

		*H. pylori-negative (n = 98)*		*H. pylori-positive (n = 101)*		*p-value*	
NLR		2.67 ± 2.35		2.91 ± 2.39		0.43	
MPV (fL)		10.18 ± 0.91		10.02 ± 0.78		0.17	
Platelet (μL)		258,247 ± 69,494		270,039 ± 79,856		0.10	
Leukocyte (μL)		7,686 ± 2,714		8,053 ± 2,541		0.99	
Neutrophil (μL)		4,722 ± 2,346		5,156 ± 2,213		0.98	
Lymphocyte (μL)		2,150 ± 826		2,280 ± 1,259		0.18	
Age		45 ± 16		48 ± 17		0.44	

The distribution of parameters, in which 98 *H. pylori-*negative patients and 57 *H. pylori*-positive patients of mild intensity according to Sydney classification are compared, is shown in Table 2.

The distribution of parameters, in which 98 *H. pylori-*negative patients and 26 *H. pylori*-positive patients of moderate intensity according to Sydney classification are compared, is shown in Table 3.

**Table 2:** **Table 2:** Comparison of *H.* pylori-negative and *H. pylori-positive* patients of mild intensity according to Sydney classification

		*H. pylori-negative (n = 98)*		*Mild H. pylori-positive (n = 57)*		*p-value*	
NLR		2.67 ± 2.35		3.21 ± 2.60		0.14	
MPV (fL)		10.18 ± 0.91		10.01 ± 0.75		0.11	
Platelet (μL)		258,247 ± 69,494		268,947 ± 73,285		0.31	
Leukocyte (μL)		7,686 ± 2,714		8,024 ± 2,418		0.69	
Neutrophil (μL)		4,722 ± 2,346		5,268 ± 2,206		0.86	
Lymphocyte (μL)		2,150 ± 826		2,097 ± 819		0.71	
Age		45 ± 16		48 ± 18		0.33	

**Table 3:** **Table 3:** Comparison of *H. pylori-negative* and *H. pylori-positive* patients of moderate intensity according to Sydney classification

		*H. pylori-negative (n = 98)*		*Moderate H. pylori-positive (n = 26)*		*p-value*	
NLR		2.67 ± 2.35		1.94 ± 0.79		0.04*	
MPV (fL)		10.18 ± 0.91		9.84 ± 0.77		0.39	
Platelet (μL)		258,247 ± 69,494		275,500 ± 68,449		0.66	
Leukocyte (μL)		7,686 ± 2,714		7,269 ± 2,731		0.93	
Neutrophil (μL)		4,722 ± 2,346		4,211 ± 1,998		0.61	
Lymphocyte (μL)		2,150 ± 826		2,215 ± 630		0.20	
Age		45 ± 16		50 ± 15		0.59	

*statistically significant (p < 0.05)

The distribution of parameters, in which 98 *H. pylori-*negative patients and 18 *H. pylori*-positive patients of severe intensity according to Sydney classification are compared, is shown in Table 4.

## DISCUSSION

*H. pylori* is a microorganism causing gastritis and gastric functional changes virtually in all infected persons; peptic ulcer in 15 to 20%, ulcer complication in 2 to 12%, and serious gastric diseases like stomach cancer in 1 to 3%, and B-cell lymphoma (mucosa associated lymphoid tissue lymphoma), a primary gastric lymphoma, in 0.1% of infected persons.^1^ The results of a meta-analysis on three prospective epidemiologic studies have shown that *H. pylori-positive* patients are exposed to four times higher cancer development risk than normal patients, and the recent decline in antrum and corpus cancer is parallel to the decline in the prevalence of *H. pylori.^5^* In a study, sedimentation fibrinogen and CRP, acute-phase reactants, were found to be high in *H. pylori*-positive patients, and a significant relationship was shown between *H. pylori* and systemic inflammatory response.^18^ The purpose of our study was to determine the changes of *H. pylori,* the most common infectious agent in the world and recognized as a carcinogen, on automated hemogram test, a simple blood test. A simple, inexpensive, and quickly concluded hemogram does not only give us values, such as hemoglobin and leukocyte but also MPV, absolute lymphocyte, and absolute neutrophil values. Moreover, NLR is a value calculated by dividing absolute neutrophil count by absolute lymphocyte count. Although studies related to platelet and MPV were present as they are closely related to the cardiovascular system, *H. pylori* causes a chronic inflammation in the gastric mucosa that prevails for years, though in varying severity, and chronic infection depends upon several factors, including bacterial resistance and host response.^19^

Comparing the *H. pylori*-positive patients and *H.* pylori-negative patients, we could not find any significant difference between them in our study in terms of NLR and MPV values. However, Farah and Khamisy-Farah^20^ found in their 2014 study that the *H. pylori*-positive patients had higher NLR values than *H. pylori*-negative patients. They also identified a parallel increase between the severity of the *H. pylori* gastritis and NLR values.^20^ In another study conducted by Jafarzadeh et al,^21^ it was established that the *H. pylori*-positive patients, independent of the bacterial cytotoxin-associated gene A (CagA) status and regardless of peptic ulcer, had more leukocyte, neutrophil, and NLR ratios in their blood than that of the *H. pylori-negative* patients. It is interesting that, in the subgroup analyses of our study, we have found lymphocyte and thrombocyte values of the *H. pylori*-positive patients within the normal range but significantly higher than that of the *H. pylori-negative* patients. Such an increase in the absolute lymphocyte values has probably avoided the finding of a higher NLR ratio. Having a limited number of severe *H. pylori* gastritis cases in our study has a restrictive effect on our study in that particular respect. In this respect, more studies should be conducted with higher number of severe *H. pylori* gastritis patients.

**Table 4:** **Table 4:** Comparison of *H. pylori-negative* and *H. pylori-positive* patients of severe intensity according to Sydney classification

		*H. pylori-negative (n = 98)*		*Severe H. pylori-positive (n = 18)*		*p-value*	
NLR		2.67 ± 2.35		3.30 ± 2.93		0.17	
MPV(fL)		10.18 ± 0.91		10.32 ± 0.84		0.81	
Platelet (μL)		258,247 ± 69,494		265,611 ± 113,397		0.02*	
Leukocyte (μL)		7,686 ± 2,714		9,276 ± 2,286		0.84	
Neutrophil (μL)		4,722 ± 2,346		6,166 ± 2,099		0.82	
Lymphocyte (μL)		2,150 ± 826		2,954 ± 2,436		0.00*	
Age		45 ± 16		46 ± 18		0.36	

*statistically significant (p < 0.05)

In our study, we found that absolute lymphocyte count in peripheral blood increased significantly as intensity of *H. pylori* increased, and this finding appears to support the study carried out by Karttunen et al.^22^ There is a very tight correlation between *H. pylori* count in the mucosa and severity of tissue inflammation.^23^ As there is no organized lymphoid tissue in the gastric mucosa, any increase in cell count associated with *H. pylori* bacteria at this site will undoubtedly be correlated with peripheral blood cells. Since *H. pylori* is not an invasive microorganism and causes low chronic antigenic stimulation, we could not determine NLR values in *H. pylori*-positive patients; however, mean NLR value was higher in *H.* pylori-positive patients than in *H. pylori-negative* patients, but was not statistically significant. We found that the reason for absence of significantly high NLR, as expected in *H. pylori*-positive patients, was the fact that absolute lymphocyte counts increased as the intensity of *H. pylori* increased and that NLR value did not rise. The increase in absolute lymphoid count in peripheral blood must be evaluated as a very important finding.

Mean platelet volume can be obtained from hemo-gram, which is a routine and easy-to-obtain indicator showing systemic inflammation. High MPV values show larger and active platelets that contribute to thrombocytic events. The MPV is a marker showing platelet volume and can be easily determined by hemogram. Larger platelets mean much more active platelets, both metabolically and enzymatically, and have been shown to contribute to proinflammatory and prothrombotic situation.^24^ Furthermore, the correlation between increases in MPV and myocardial infarction and mortality has been shown.^15^ The intensity of systemic inflammation determines size and volume of platelets. Some epidemiologic studies have shown the correlation between *H. pylori* and coronary artery disease.^15^ In a study that we previously carried out in relation to MPV in the past, we found statistically lower MPV values in ulcerative colitis (UC), and construed that lower MPV values might reflect UC disease activity.^25^ In this study, we did not find any change in MPV in *H. pylori*-positive patients, and this may be due to absence of a significant inflammatory effect like active UC and presence of a chronic low-grade inflammatory event. In light of this information, there is no consensus in the literature about MPV and inflammation. There are studies on ITP in *H. pylori*-positive patients, and a decline in platelets is expected in *H. pylori*-positive patients. There are studies performed with 9.25 and 9.70 fL for MPV, indicating that it predicts cardiovascular disease.^26^ In our study, only patients with antral gastritis on endoscopy were divided into four groups as *H. pylori*-negative and mild, moderate, and severe *H. pylori*-positive according to intensity, and no significant difference was found between these four groups in terms of MPV. This is consistent with the previous studies in the literature.^14^ However, in the comparison of these four groups, statistically higher platelet counts were found in severe *H. pylori*-positive patients than in *H. pylori*-negative patients on hemogram. This finding strengthens the correlation between cardiovascular, notably coronary artery disease, and presence of *H. pylori* infection.

## CONCLUSION

There were no differences in terms of NLR and MPV between total *H. pylori*-negative patients and *H. pylori-*positive patients. A moderate increase in the intensity of *H. pylori* does not lead to a significant change in MPV measured by hemogram; however, it gives rise to a statistically significant fall in NLR, which, we estimate, is due to the increase in absolute lymphocyte count on hemogram. Presence of severe *H. pylori*-positive intensity leads to a statistically significant increase in peripheral blood lymphocytes and platelets compared with *H. pylori-*negative patients.
